# Clinical evaluation of a wearable sensor for mobile monitoring of respiratory rate on hospital wards

**DOI:** 10.1007/s10877-021-00753-6

**Published:** 2021-09-02

**Authors:** Kristiina Järvelä, Panu Takala, Frederic Michard, Leena Vikatmaa

**Affiliations:** 1 Research & Development, GE Healthcare, Helsinki, Finland; 2 MiCo, Denens, Switzerland; 3 Department of Anesthesia, Intensive Care and Pain Medicine, Helsinki University Hospital and University of Helsinki, Helsinki, Finland

**Keywords:** Ward monitoring, Wireless monitoring, Remote monitoring, Wearable sensor, Respiratory frequency, Thoracic impedance

## Abstract

A wireless and wearable system was recently developed for mobile monitoring of respiratory rate (RR). The present study was designed to compare RR mobile measurements with reference capnographic measurements on a medical-surgical ward. The wearable sensor measures impedance variations of the chest from two thoracic and one abdominal electrode. Simultaneous measurements of RR from the wearable sensor and from the capnographic sensor (1 measure/minute) were compared in 36 ward patients. Patients were monitored for a period of 182 ± 56 min (range 68–331). Artifact-free RR measurements were available 81% of the monitoring time for capnography and 92% for the wearable monitoring system (p < 0.001). A total of 4836 pairs of simultaneous measurements were available for analysis. The average reference RR was 19 ± 5 breaths/min (range 6–36). The average difference between the wearable and capnography RR measurements was − 0.6 ± 2.5 breaths/min. Error grid analysis showed that the proportions of RR measurements done with the wearable system were 89.7% in zone A (no risk), 9.6% in zone B (low risk) and < 1% in zones C, D and E (moderate, significant and dangerous risk). The wearable method detected RR values > 20 (tachypnea) with a sensitivity of 81% and a specificity of 93%. In ward patients, the wearable sensor enabled accurate and precise measurements of RR within a relatively broad range (6–36 b/min) and the detection of tachypnea with high sensitivity and specificity.

## Introduction

Unexpected deaths on hospital wards remain all too common [1–3]. In a UK national audit study, among 23,554 adult in-hospital cardiac arrests, more than half (57%) occurred on the wards and only 5% in the intensive care unit (ICU) [4]. In the large (> 46,000 patients) EUSOS study done in 28 European countries, most surgical patients (73%) who died before hospital discharge were not admitted to critical care at any stage after surgery [5]. Importantly, most ward patients do not suddenly deteriorate. Vital signs are often abnormal, or trending toward abnormal range, hours before cardiac arrest or ICU transfer [6]. But healthcare workers may only suddenly notice this is happening because spot-checks are usually done on a 4–8 h interval. Finding patients before they rapidly deteriorate and suffer a serious adverse event might be the next major opportunity to improve patient safety [1–3, 7].

Vital signs classically spot-checked in ward patients include heart rate, blood pressure, respiratory rate (RR), oxygen saturation and temperature. Several studies have suggested that RR manual counting is often inaccurate, when not simply neglected [8–11]. This is a paradox because studies have also shown that RR is a key variable for the early detection of clinical deterioration. In a nested case–control study including 440 ward patients, RR had a better predictive value of cardiac arrest than heart rate, systolic blood pressure and temperature [6]. In a recent study including > 260,000 ward patients and using machine learning methods for predicting clinical deterioration [12], RR had the highest “weight” in the predictive algorithm followed by heart rate, systolic blood pressure, temperature and oxygen saturation. In line with these observations, the National Institute for Health and Care Excellence in the UK stated that “RR is the best marker of a sick patient and is the first observation that will indicate a problem or deterioration in condition” (https://www.nice.org.uk/guidance/CG50).

The current rise of wireless and wearable sensors creates the opportunity for continuous monitoring in traditionally unmonitored settings [13]. Although the number of wearable sensors is quickly growing, independent validation studies done in real life conditions remain scarce [14]. Studies have reported significant but often weak relationships between RR measurements from wearable adhesive patches and other methods [10, 15–18].

**Fig. 1:**
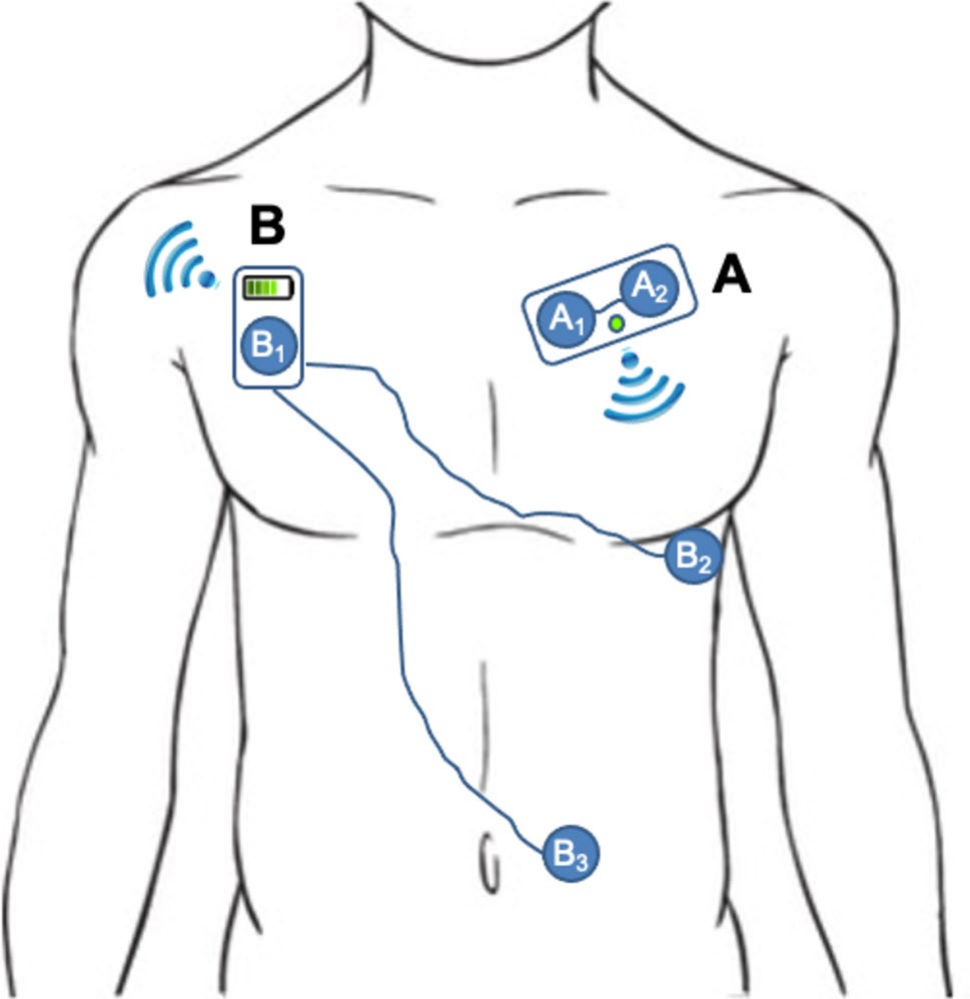
Principles of RR measurements from a 2 electrodes patch with a disposable battery (**A**) and from the new 3 electrodes sensor with a reusable battery (**B**). With A, changes in thoracic impedance are recorded between A1 and A2 when patients are breathing. With B, larger changes in thoracic impedance are recorded between B1 and B2 and between B1 and B3 (dual vector approach)

Respiration induces changes in electrical thoracic impedance that are widely used, via skin surface electrodes, to monitor RR in ICU patients. This method is known as impedance pneumography. An untethered impedance pneumography system was recently developed for mobile RR monitoring in ward patients (Fig. 1). Therefore, we designed the present study to compare RR measurements done with the new wireless system and the reference tethered capnography technique.

## Methods

This is a prospective comparison study done in Finland at the Helsinki University Hospital (HUS). The study was approved by the ethical committee of HUS (#HUS/3008/2018 approved on July 04, 2016) and written informed consent was obtained for all patients.

We studied adult patients admitted in the Emergency, Telemetry (cardiac and post-cardiac surgery) or Thoracic surgical ward. These wards were selected for the study because our research nurses had access to them and because we were expecting to record more often abnormal RR values in these “sub-acute” wards than in general hospital wards. All patients had a nasal cannula connected to a specific capnography monitor (CARESCAPE Patient Monitor B450, GE Healthcare) measuring RR continuously from the CO_2_ waveform. All patients were also monitored continuously with the new wireless sensor. This wireless sensor measures changes in thoracic impedance recorded by 2 thoracic and 1 abdominal electrode (Fig. 1). These electrodes are connected wirelessly to a smartphone-like mobile monitoring device analyzing the signals and transmitting the information to a central station. The determination of RR with the new sensor is based on evaluating changes in thoracic impedance between the skin electrodes during respiratory movements. Changes in thoracic impedance are quantified using 2 channels or a “dual vector” approach (between electrodes B1 and B2, and electrodes B1 and B3 on Fig. 1). Further details are available in the patent application (WO2019241362A1). Measurements from both sources were averaged over one-minute periods.

We compared RR measurements by calculating the mean ± SD of the differences between the two methods and presented the results using the Bland–Altman method. To assess the clinical relevance of our findings, we performed an error grid analysis, as previously proposed [16]. The error grid analysis enables a risk level to be assigned to each pair of RR values. We calculated the proportion of measurements in risk zones A–E with A indicating no risk, B low risk, C moderate risk, D significant risk, and E dangerous risk for the patient due to the risk of wrong clinical interventions because of measurement errors. Finally, we quantified the sensitivity and specificity of the new wearable sensor to detect tachypnea, as defined in the National Early Warning Score (RR > 20 breaths/min).

## Results

Forty consecutive adult ward patients were enrolled in the study. Four patients were excluded from the final analysis due to technical problems (3 patients with the capnographic sensor and 1 patient with the wearable sensor). Seventeen patients were studied on the Emergency ward, 14 on the Telemetry ward, and 5 on the Thoracic surgical ward. Four patients required supplemental oxygen. Main characteristics of the study population are summarized in Table 1.

**Table 1:** Main characteristics of the study population

Mean age, yrs (range)	58 (22–87)
Gender, Male/Female	22/14
Underlying medical conditions	
Diabetes	8
Cancer	7
Hypertension	5
Atrial fibrillation	5
Chronic obstructive pulmonary disease	2
Obstructive sleep apnea	2
Chronic heart failure	2
Chronic respiratory failure	1
Coronary artery disease	1
Main reason for hospital admission	
Postoperative cardiac surgery	10
Sepsis	7
Postoperative thoracic surgery	5
Pneumonia	4
Pulmonary embolism	2
Mesenteric ischemia	1
Arrhythmia	1
Anemia	1
Myocardial infarction	1
Pleural effusion	1
Other	3

**Fig. 2:**
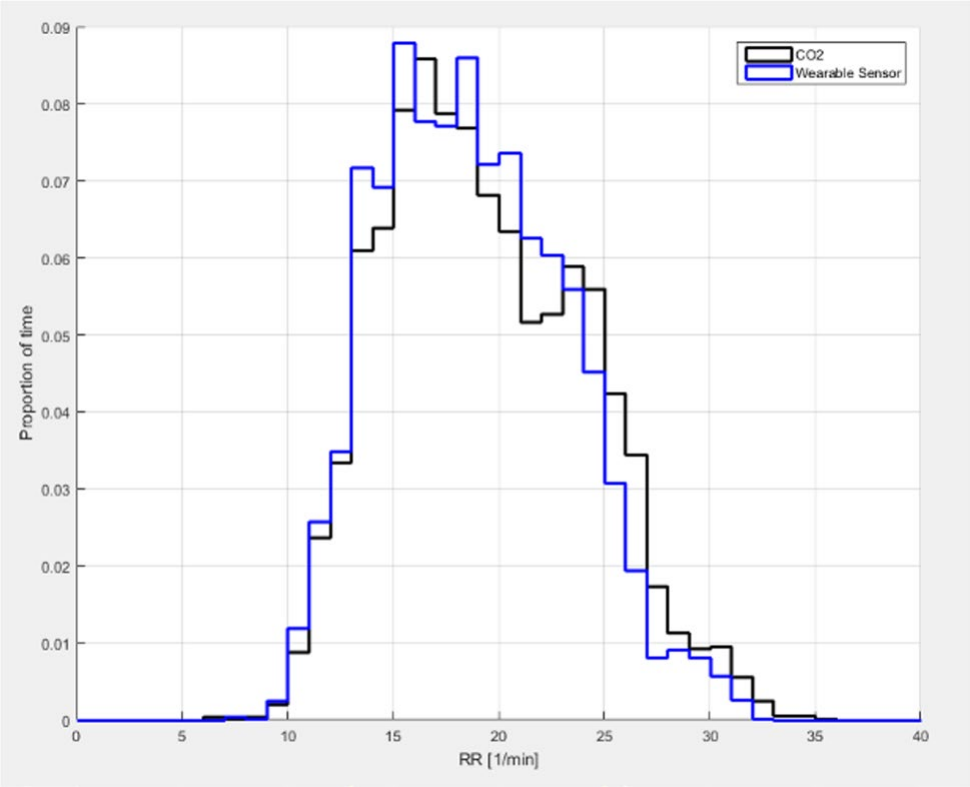
Range and proportions of RR values recorded with capnography (CO2) and the wearable sensor in 36 ward patients

**Fig. 3:**
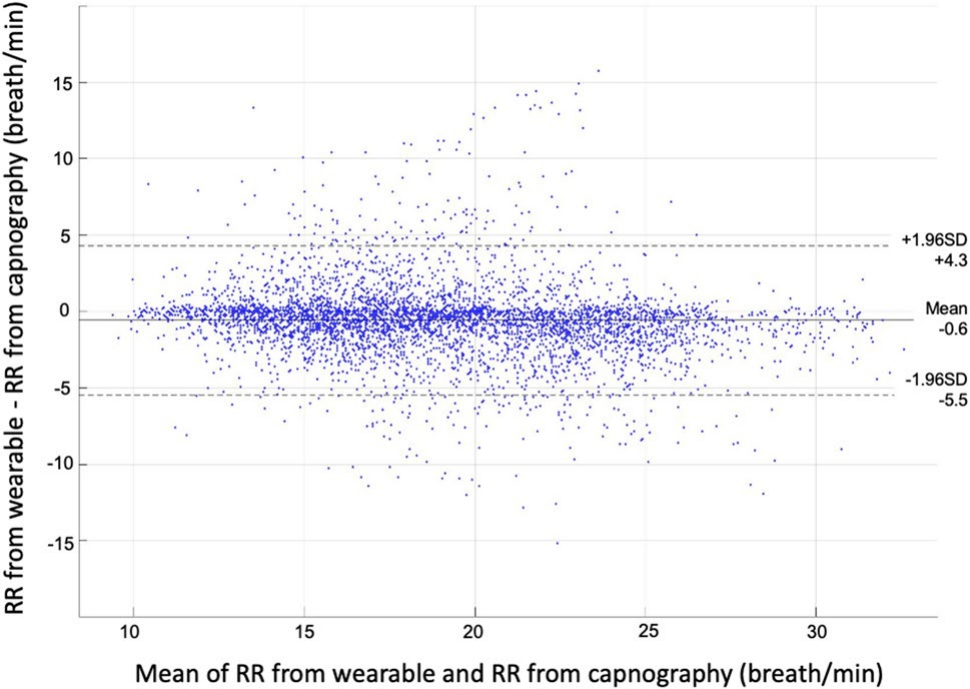
Bland & Altman comparison graph

**Fig. 4:**
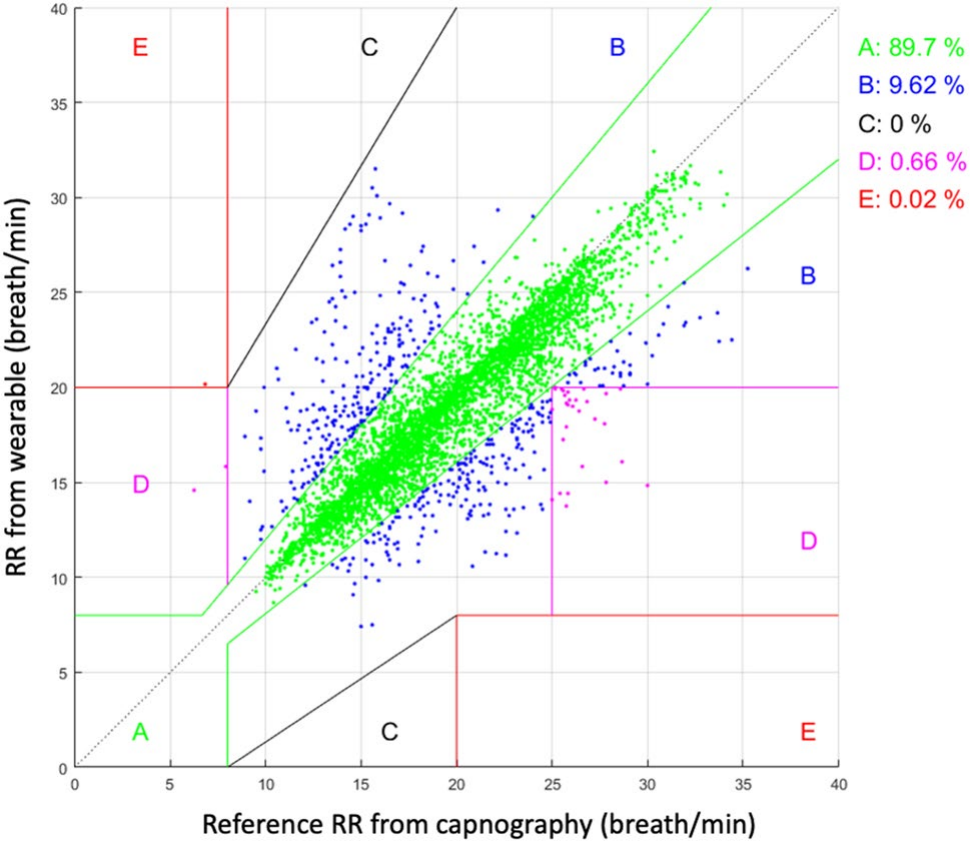
Clarke’s Error grid analysis

Patients were monitored for a period of 182 ± 56 (range 68–331) minutes. Artifact-free RR measurements were available 81% of the monitoring time for capnography and 92% for the wearable monitoring system (p < 0.001). A total of 4836 pairs of simultaneous measurements were available for analysis. The average reference RR was 19 ± 5 breaths/min (range 6–36) (Fig. 2). When comparing both methods, the difference between the wearable and the capnography RR measurements was − 0.6 ± 2.5 breaths/min (Fig. 3). The bias between the two methods was not influenced by RR (Fig. 3). The error grid analysis showed that the proportions of RR measurements done with the wearable system were 89.7% in zone A (no risk), 9.6% in zone B (low risk) and < 1% in zones C, D and E (moderate, significant and dangerous risk) (Fig. 4). The wearable method detected RR values > 20 (tachypnea) with a sensitivity of 81% and a specificity of 93%.

## Discussion

Our study suggests that monitoring RR with the new wearable sensor is feasible in ward patients, and accurate when compared to capnography measurements.

Capnography sensors detect expired CO_2_ and are the reference to measure RR. On hospital wards, they provide early warning of respiratory depression, and are more effective than pulse oximetry to detect hypoventilation and/or apnea in patients receiving supplemental oxygen [19, 20]. In this respect, they have been recommended to monitor patients receiving opioids during the postoperative period [21]. However, they are part of tethered monitoring systems with nasal prongs easily dislodged and often poorly tolerated by wide-awake patients, such as those developing hypoxemic respiratory failure or sepsis on the wards. A piezo electric sensor detecting RR and heart rate through the mattress has been proposed as an alternative to capnography for the early detection of clinical deterioration [22]. Brown et al. [23] monitored medico-surgical inpatients with this contact-free sensor and reported a significant decrease in the number of calls for cardiac arrest. However, although patients are not tied to the piezo-electric sensor, they have to remain in contact with their mattress to ensure continuous monitoring. Physical movement is useful to prevent thrombotic complications and bedsores in medical and surgical settings. Both early mobilization and physiotherapy are key elements of enhanced recovery after surgery (ERAS) programs. Therefore, wireless and wearable sensors are highly desirable to make continuous RR monitoring a reality in mobile patients [24]. Skin surface electrodes, required to detect respiratory-induced changes in thoracic impedance, have the advantage to be easy to use for nurses and comfortable for patients.

Accuracy is a requirement when monitoring vital signs on the wards, first and foremost to guarantee patient safety, but also to decrease false alarms and prevent alarm fatigue. Published validation studies of new remote monitoring systems remain scarce, and head-to-head comparisons of several sensors in the same patient population are almost inexistent. Breteler et al. [16] recently compared four different monitoring systems specifically designed for ward patients, including a piezo electric bed sensor, an acoustic neck sensor, and two thoracic adhesive patches, one estimating RR from changes in thoracic impedance, and the other one from an accelerometer and ECG respiratory changes. The percentage of measurements in the error grid zones A&B (no and low risk zones) were 97%, 96%, 92% and 77%, respectively. These findings highlight the performance of the dual vector sensor tested in the present study since > 99% of our measurements were in zones A&B (Fig. 4). However, we cannot claim superiority over other solutions since the evaluations have not been done at the same time in the same patient population.

The reliability of RR measurements with impedance pneumography depends both on the number and the correct positioning of the electrodes [25]. In particular, when electrodes are very close to each other’s, the magnitude of respiration-induced changes in thoracic impedance may be very small and hence prone to error measurements (Fig. 1, system A). Several studies have tested the accuracy of wearable adhesive patches containing 2 adjacent electrodes to monitor RR on the wards and have yielded somewhat disappointing results [10, 16, 17]. These findings could be explained, at least in part, by the design of these wearables, namely the short distance between the electrodes and the recording of a single impedance signal (between 2 electrodes). The wireless sensor tested in the present study measures changes in thoracic impedance recorded by electrodes which are > 20 cm apart. In addition, it computes two impedance signals simultaneously (dual vector approach) (Fig. 1, system B). This may explain why we found it to be as accurate and precise than capnography, and able to detect tachypnea with high sensitivity and specificity.

Our study has several limitations. First, we monitored patients over a relatively short period of time (a few hours) so that future studies will have to confirm similar results can be obtained when patients are monitored several days. Second, a dedicated research nurse was continuously present in the room to fix any technical issues and to help patients in case of disconnection or during ambulation. Additional limitations and artifacts may be observed when patients are left alone with their monitoring system, particularly with the capnography sensor (e.g. disconnection-reconnection when patients are leaving their bed). Third, a proprietary wireless connectivity protocol running on a protected medical grade bandwidth was developed to prevent disruptions which are common with classical Bluetooth based wireless systems. We did not record any disruption problems during the present evaluation, but we studied one patient at a time. Further testing is desirable to confirm the robustness of the connectivity protocol when several patients are monitored at the same time and in the same room. Fourth, we did not record enough low RR values to test the sensitivity and specificity of the new sensor to detect bradypnea or apnea. Finally, other studies are warranted to confirm the new wearable monitoring system enables an earlier detection of complications when compared to intermittent spot-checks and to investigate the possible impact on clinical outcomes.

## Conclusion

In ward patients, the new dual vector wireless and wearable sensor enabled accurate and precise measurements of RR within a relatively broad range (6–36 b/min) and the detection of tachypnea with high sensitivity and specificity. It has potential to facilitate continuous monitoring of RR in ambulatory ward patients and to detect clinical deterioration at an early stage.
